# The Impact of Diagnostic Laparoscopy on Upstaging Patients with Siewert II and III Gastroesophageal Junction (GEJ) Cancer

**DOI:** 10.1245/s10434-024-15862-0

**Published:** 2024-11-06

**Authors:** Nathan J. Alcasid, Deanna Fink, Kian C. Banks, Cynthia J. Susai, Katherine Barnes, Rachel Wile, Angela Sun, Ashish Patel, Simon Ashiku, Jeffrey B. Velotta

**Affiliations:** 1 Department of General Surgery, University of California, San Francisco-East Bay, Oakland, CA USA; 2 Division of Thoracic Surgery, Department of Surgery, Kaiser Permanente Northern California, Oakland, CA USA; 3 Division of Research, Kaiser Permanente Northern California, Oakland, CA USA; 4 Department of Clinical Science, Kaiser Permanente Bernard J. Tyson School of Medicine, Pasadena, CA USA; 5 Department of Surgery, University of California, San Francisco, San Francisco, CA USA

**Keywords:** Siewert classification, Gastroesophageal junction (GEJ) tumors, Neoadjuvant therapy

## Abstract

**Background:**

The efficacy of routine diagnostic laparoscopy with cytologic evaluation for gastroesophageal junction (GEJ) cancer is variable with no set guidelines. We hypothesize that findings from diagnostic laparoscopy in Siewert II and III GEJ tumors may differ, where routine diagnostic laparoscopy with washings yields low upstaging results in Siewert II compared with Siewert III tumors.

**Patients and Methods:**

We reviewed patients with Siewert II/III GEJ cancer from 2012 through 2022 within our integrated health system. Chi-squared, Fisher’s exact, and two-sample Wilcoxon rank-sum tests were utilized. The outcomes measured include likelihood of upstaging, cytology positivity, times to chemotherapy and surgery, and 5-year mortality using a multivariable Cox regression model.

**Results:**

Of 265 patients with Siewert II diagnosis, 116 patients underwent a diagnostic laparoscopy while 149 patients did not. Median time to chemotherapy initiation and definitive surgery were increased among patients with diagnostic laparoscopy, with no difference observed in 5-year survival. For patients with Siewert II and III with a diagnostic laparoscopy, 5% of Siewert II were upstaged, compared with 17% of Siewert III (*p* = 0.025). Obtaining cytologic washings alone were less likely to be upstaged compared with receiving a biopsy with or without washings (5.2% vs. 17.3%, *p* = 0.039), and those with Siewert II were less likely than Siewert III to be upstaged after diagnostic laparoscopy (5.2% vs. 17.4%, *p* = 0.025).

**Conclusions:**

Routine diagnostic laparoscopy yields a low upstaging rate in Siewert II GEJ adenocarcinomas (AC) while delaying treatment with no improvement on mortality. Expediting definitive surgery with selective biopsy in lieu of diagnostic laparoscopy may improve oncologic outcomes.

Gastroesophageal junction (GEJ) cancer has poor prognosis, with a 5-year survival of less than 25%, largely due to the aggressive nature of the tumor and its late-stage presentation.^1–3^ Though incidence rates of certain types of esophageal and gastric cancers have declined, such as squamous cell carcinoma (SCC) of the esophagus and distal gastric carcinomas, epidemiologic studies have demonstrated an unprecedented rise in the incidence of proximal gastric and GEJ adenocarcinomas (AC).^3,4^

The Siewert classification was created in an attempt to better classify and diagnose GEJ AC for oncologic staging with surgical implications.^5^ Tumor classification is based on proximity to the GEJ: Siewert I tumors are located in the distal esophagus with approximately 5 cm extension proximally from the GEJ, Siewert II tumors are located at the GEJ, and Siewert III tumors are located in proximal stomach with approximately 5 cm extension distally from GEJ.^1^ With these anatomical definitions in place, the tumor, node, metastasis (TNM) staging classification of the 8th edition of American Joint Committee on Cancer (AJCC) stages Siewert II tumors as esophageal tumors, despite no consensus guidelines regarding the optimal surgical approach, that is, esophagectomy or total gastrectomy.^4,6^ Tumor location also has implications regarding nodal metastasis, with Siewert I tumors typically involving upper mediastinal lymph nodes and Siewert II/III more frequently involving gthe lower mediastinum in addition to the intraabdominal celiac axis.^7,8^ Over the past decade, more than half of GEJ tumors were diagnosed as Siewert II.^9^ Despite the increasing incidence and associated high mortality, there remains little consensus guidelines regarding the optimal preoperative and operative management. In addition, the role and efficacy of routine diagnostic laparoscopy with cytologic evaluation regarding stage migration for gastroesophageal junction (GEJ) cancer is variable and not as well defined in the literature compared with primary gastric cancers.

In patients with diagnosed Siewert II and III tumors, common staging workup typically includes a combination of preoperative computed tomography (CT) scan, positron emission tomography (PET) scan, and endoscopic ultrasound (EUS) with fine-needle aspiration (FNA).^10,11^ According to the National Comprehensive Cancer Network (NCCN) guidelines, diagnostic laparoscopy is “optional” for patients with GEJ tumors without evidence of metastasis on preoperative imaging.^11–13^ The addition of FNA with advances in EUS have greatly increased the diagnostic sensitivity compared with prior CT and/or FNA alone.^14,15^ It has been previously demonstrated that EUS-FNA had 90% accuracy as diagnostic laparoscopy in identifying nodal disease in those with esophageal adenocarcinoma.^16^ Consequently, patients undergoing diagnostic laparoscopy are potentially delayed in receiving earlier multimodal treatment with neoadjuvant chemotherapy or chemoradiation, which has consistently demonstrated increased R0 resection rates through downstaging, decreasing the number of involved lymph nodes and improving long-term survival compared with surgery alone.^17^ We hypothesize that screening diagnostic laparoscopy is an unnecessary step in the oncologic management in Siewert II GEJ cancer and will allow us to initiate chemotherapy and definitive surgical intervention sooner, which may allow for better outcomes. Additionally, we sought to determine whether treatment algorithms should differ between Siewert II and III tumors, with the belief that routine diagnostic laparoscopy with washings yields low upstaging results in Siewert II compared with Siewert III tumors.

## Patients and Methods

### Study Population

We performed a retrospective review of all adult patients (≥ 18 years) with Siewert II and Siewert III GEJ cancer from 1 January 2012 through 30 June 2022 within our integrated healthcare delivery system, which serves as an esophageal cancer tertiary referral center. Patients with Siewert I and gastric cancers were excluded. This study was approved as observational and data-only study by the Kaiser Foundation Research Institute’s institutional review board with a waiver of consent because of minimal risk with de-identified data.

### Study Design

At our institution patients are diagnosed through a combination of CT, PET, and EUS-FNA. Surgical resection is performed via a minimally invasive Ivor–Lewis or transhiatal esophagectomy. We chart reviewed all patients diagnosed with Siewert II and Siewert III to compare patients who underwent diagnostic laparoscopy as part of their oncologic staging with those who did not receive a diagnostic laparoscopy. Baseline characteristics, comorbidities, and cancer stages of patients with Siewert II and III GEJ cancer between those who underwent diagnostic laparoscopy and those who did not were compared. Patient and clinical characteristics associated with “upstaging” or stage migration were also evaluated. The effect of cytologic washings, biopsy, and/or a combination of both was also evaluated in patients who underwent a diagnostic laparoscopy versus those who did not. Age, race/ethnicity, sex, body mass index (BMI) at diagnosis, Charlson Comorbidity Index (CCI) score, smoking history, and history of alcohol abuse were obtained from electronic databases.

### Outcomes

Our primary outcomes focused on patients with Siewert II who underwent diagnostic laparoscopy versus those who did not and included time to chemotherapy initiation, time to surgery, and mortality at 5 years. Our secondary outcomes focused on the effects of cytologic staging among Siewert II and Siewert III GEJ tumors after diagnostic laparoscopy, and include cytology positivity, likelihood of upstaging, and time to surgery. “Time” was defined as days from initial biopsy confirmed malignancy to the date of interest (chemotherapy initiation or surgery). Upstaging is defined as stage migration from clinical to pathological stage.

### Statistical Analysis

For both our primary and secondary outcomes, categorical baseline characteristics and receipt of chemotherapy and definitive surgery were compared using chi-squared tests or Fisher’s exact tests for smaller populations. Non-normally distributed continuous variables including age at diagnosis and the outcome variables for median time to chemotherapy and time to definitive surgery were compared using nonparametric Wilcoxon rank-sum tests.

Kaplan–Meier and Cox regression analyses were performed for one of the primary outcomes, 5-year survival among patients with Siewert II, comparing those without versus with a diagnostic laparoscopy. Patients were followed from date of diagnosis until either (1) death, (2) the end of the 5-year period, or (3) last date of available mortality data. Patients who were lost to follow-up before the end of the 5-year period were censored at their last confirmed membership date. The proportional hazards assumption was assessed with log–log graphs and Schoenfeld residuals. All analyses were conducted in SAS version 9.4 (SAS Institute). Statistical significance was set to *p* < 0.05. All statistical tests were two-tailed.

## Results

There were 282 adult patients identified with a Siewert II diagnosis during the study period from 1 January 2012 to 30 June 2022. A total of 17 patients were excluded from the study (6.0%) mostly due to palliative care treatment or loss of follow-up. Of the 265 eligible patients, 116 patients underwent a diagnostic laparoscopy for oncologic staging prior to chemotherapy and definitive surgery, while 149 patients did not. There were no statistically significant differences in the baseline patient characteristics between patients with Siewert II who did and did not receive a diagnostic laparoscopy (age, race/ethnicity, sex, BMI, Charlson Comorbidity Index score, smoking history, and history of alcohol abuse). Patients were most often Stage III at their time of diagnosis (52% among all patients with Siewert II). Patients who received a diagnostic laparoscopy were more likely to be stage III at time of diagnosis, compared with those who did not receive a diagnostic laparoscopy (67% vs. 40%, respectively) (Table 1).

**Table 1:** Baseline characteristics of the 265 patients diagnosed with Siewert II

Variables	Total (*N* = 265)	No diagnostic laparoscopy (*N* = 149)	Diagnostic laparoscopy (*N* = 116)	*p* value
Age				0.191^Ɨ^
Median (IQR)	67.0 (60.0–74.0)	68.0 (61.0–76.0)	66.0 (58.0–73.0)	
Race/ethnicity				0.154*
White	188 (70.9)	106 (71.1)	82 (70.7)	
Black/African American	8 (3.0)	2 (1.3)	6 (5.2)	
Hispanic	31 (11.7)	22 (14.8)	9 (7.8)	
Asian/Pacific Islander	31 (11.7)	15 (10.1)	16 (13.8)	
Other/unknown	7 (2.6)	4 (2.7)	3 (2.6)	
Sex				0.606
F	40 (15.1)	21 (14.1)	19 (16.4)	
M	225 (84.9)	128 (85.9)	97 (83.6)	
BMI				0.106
< 25	81 (30.6)	41 (27.5)	40 (34.5)	
25.0–29.9	113 (42.6)	72 (48.3)	41 (35.3)	
≥ 30	71 (26.8)	36 (24.2)	35 (30.2)	
Stage at biopsy				< 0.001
I	15 (5.7)	13 (8.7)	2 (1.7)	
II	28 (10.6)	23 (15.4)	5 (4.3)	
III	138 (52.1)	60 (40.3)	78 (67.2)	
IVA	40 (15.1)	15 (10.1)	25 (21.6)	
IVB	44 (16.6)	38 (25.5)	6 (5.2)	
CCI score (inpatient and outpatient)				0.779
0–3	158 (59.6)	87 (58.4)	71 (61.2)	
4–5	42 (15.8)	23 (15.4)	19 (16.4)	
6+	65 (24.5)	39 (26.2)	26 (22.4)	
History of smoking				0.209
Yes	32 (20.4)	15 (16.9)	17 (25.0)	
Former smoker	125 (79.6)	74 (83.1)	51 (75.0)	
No/never	108 (40.8)	60 (40.3)	48 (41.4)	
History of alcohol abuse				0.733*
Yes	8 (3.0)	4 (2.7)	4 (3.4)	
No	257 (97.0)	145 (97.3)	112 (96.6)	

*Used Fisher’s exact test due to small cell sizes and chi-squared test for all other categorical variables

^Ɨ^Used Wilcoxon two-sample non-parametric test to compare median age

**Fig. 1:**
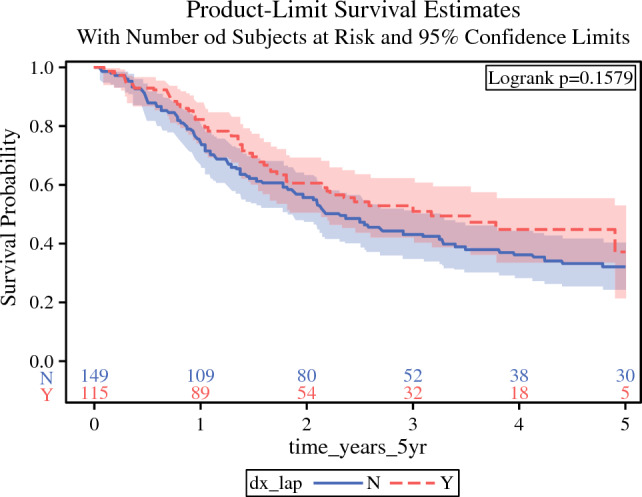
Kaplan–Meier curve modeling diagnostic laparoscopy versus no diagnostic laparoscopy on 5-year survival in Siewert II CA (*p* = 0.158)

The median time to chemotherapy initiation (measured from day of diagnosis) was increased in patients who had undergone diagnostic laparoscopy [42 (95% CI 35–49) days] compared with those who did not [37 (95% CI 28–47) days] (*p* = 0.002) (Table 2). The median time to definitive surgery was also increased in patients who had undergone diagnostic laparoscopy [135 (95% CI 119–155) days] compared with those who did not [125 (95% CI 107–149) days] (*p* = 0.045) (Table 2). There was no significant difference in 5-year survival between those who did not undergo a diagnostic laparoscopy compared with those who did, both in bivariate Kaplan–Meier analysis (*p* = 0.158) and in a multivariable Cox regression model adjusting for age, clinical stage, and Charlson Comorbidity Index (CCI) category (aHR 1.33, 95% CI 0.95–1.88, *p* = 0.100). A statistically significant 5-year survival difference was observed for clinical staging (*p* < 0.001) and age (*p* = 0.014) (Fig. 1 and Table 3). Visually, a log–log plot indicated parallel lines for diagnostic laparoscopy category, supporting the assumption of constant hazard ratios over time. Additionally, the Schoenfeld residuals test did not reveal any significant departures from proportionality (global *p* = 0.87).

**Table 2:** Association of diagnostic laparoscopy on outcomes in 265 patients diagnosed with Siewert II

Variables	Total (*N* = 265)	No diagnostic laparoscopy (*N* = 149)	Diagnostic laparoscopy (*N* = 116)	*p* value
Chemo (Y/N)				0.017
Y	238 (89.8)	128 (85.9)	110 (94.8)	
N	27 (10.2)	21 (14.1)	6 (5.2)	
Time to chemo (in days, first cancer dx dt as baseline)				0.002*
Median (IQR)	40.0 (32.0–48.0)	37.0 (28.0–46.5)	42.0 (35.0–49.0)	
Definitive surgery (Y/N)				< 0.001
Y	153 (57.7)	68 (45.6)	85 (73.3)	
N	112 (42.3)	81 (54.4)	31 (26.7)	
Time to definitive surgery (in days, first cancer dx dt as baseline)				0.045*
Median (IQR)	131.5 (114.5–153.0)	124.5 (107.0–149.0)	134.5 (119.0–154.5)	

*Used Wilcoxon two-sample non-parametric test to compare median times with chemotherapy and surgery. Chi-squared test used for the categorical variables

**Table 3:** Multivariable Cox regression analysis of 5-year survival from the date of diagnosis among patients diagnosed with Siewert II who did not have a diagnostic laparoscopy versus those who did have a diagnostic laparoscopy

Variable	Adjusted hazard ratio (95% CI)	*p* value
Diagnostic laparoscopy		
Y	Reference	
N	1.33 (0.95–1.88)	0.100
Clinical stage at biopsy	1.68 (1.35–2.12)	< 0.001
Age (continuous)	1.02 (1.00–1.04)	0.014
Charlson comorbidity index		
0–3	Reference	
4–5	0.97 (0.59–1.55)	0.915
6+	1.20 (0.81–1.76)	0.358

When comparing patients with Siewert II and III who underwent a diagnostic laparoscopy (*n* = 162), 5% of Siewert II were upstaged compared with 17% of Siewert III (*p* = 0.025) (Table 4). Upstaging was associated with a median 55-day delay in time to surgery (*p* = 0.035) and a decreased rate of definitive surgery (*p* < 0.001) (Table 4). Patients who received cytologic washings alone were less likely to be upstaged compared with patients who received biopsy with or without washings (5.2% vs. 17.3%, *p* = 0.039) (Table 4).

**Table 4:** Association between upstaging and outcomes in 162 patients diagnosed with Siewert II and III who underwent diagnostic laparoscopy

Variables	Total (*N* = 162)	Not upstaged (*N* = 148)	Upstaged (*N* = 14)	*p* value
Siewert classification				0.025
2	116	110 (94.8)	6 (5.2)	
3	46	38 (82.6)	8 (17.4)	
Washing or biopsy				0.039
Washings alone	97	92 (94.8)	5 (5.2)	
Biopsy with or w/o washings	52	43 (82.7)	9 (17.3)	
Neither	13	13 (100.0)		
Definitive surgery				< 0.001
Y	111 (68.5)	109 (98.2)	2 (1.8)	
N	51 (31.5)	39 (76.5)	12 (23.5)	
Time to definitive surgery (in days, first cancer dx dt as baseline)				0.035*
Median (IQR)	138.0 (119.0–156.0)	137.0 (119.0–155.0)	192.0 (188.0–196.0)	

*Used Wilcoxon two-sample non-parametric test to compare time to definitive surgery. Chi-squared tests used for all categorical variables

## Discussion

This is the first known study of its kind comparing outcomes in patients with GEJ Siewert II and III tumors undergoing staging diagnostic laparoscopy. As expected, patients who underwent diagnostic laparoscopy had longer times to initiate chemotherapy and definitive surgery. As current literature is unable to amalgamate a consensus perioperative and operative approach to all Siewert II tumors, we believe our study demonstrates a plausible and distinct approach to expedite care to chemotherapy and definitive surgery in patients with Siewert II compared with Siewert III tumors.

Current NCCN guidelines recommend “consideration” of staging diagnostic laparoscopy for GEJ tumors with evidence of locoregional disease after radiographic and EUS staging while some advocate for tumors > T1 and/or T3/T4 diagnosed on EUS.^11,18^ EUS has been well studied and is now considered “standard of care” for the staging algorithm for esophageal cancer at most tertiary centers, with its ability to predict survival having an 85% accuracy for detecting T stage and 75% accuracy detecting N stage.^14^ The addition of EUS with FNA increases its overall accuracy and sensitivity to determine not only T stage, but also and more importantly, locoregional disease including celiac axis lymph nodes.^19^ Compared with patients who underwent a staging diagnostic laparoscopy, EUS-FNA was found to have a nodal staging accuracy of 90%.^16^ Though diagnostic laparoscopy has the benefit of detecting distant metastatic lesions not found on EUS or PET, its inability to detect mediastinal lymph node basins, accurately define T stage, and its high operating costs lends itself to not be particularly cost-effective as an independent procedure.^16,20^

Similar to prior studies, the majority of our patients with Siewert II tumors were stage III.^16^ Despite advanced disease, preoperative staging with EUS-FNA and CT/PET alone has been shown to be able to sufficiently direct preoperative therapy and change management in up to 77% of cases.^19^ We believe that preoperative staging protocols are sufficient with EUS-FNA, in addition to CT and PET evaluation, and foregoing a staging diagnostic laparoscopy to expedite time to chemotherapy and definitive surgery.

Though diagnostic laparoscopy may have superiority over EUS-FNA regarding distant disease, its clinical relevance regarding changing outcomes has not been well studied. Current NCCN guidelines recommend multimodality therapy including neoadjuvant chemoradiation or perioperative chemotherapy for locally advanced GEJ tumors.^11,21^ As EUS-FNA is the gold standard in determining locoregional staging, it has the largest implications in guiding preoperative therapy, particularly neoadjuvant chemotherapy, which has been known to vastly improve outcomes compared with surgery alone.^16,17^ This is particularly strengthened with the addition of CT and PET in detecting distant disease aiding in surgical planning.^14^ Though diagnostic laparoscopy may have improved accuracy in detecting occult metastatic disease, we found that its clinical use had no significant changes in outcomes. Patients with Siewert II tumors who underwent a staging diagnostic laparoscopy had no significant difference in mortality at 5 years on Cox regression compared with those whose staging relied on CT, PET, and EUS-FNA. Thus, the diagnostic value of a staging diagnostic laparoscopy may be overstated, with no clinical benefit in mortality. Our findings suggest that the potential staging algorithm would consist of preoperative imaging with PET/CT with the addition of EUS-FNA to guide neoadjuvant therapy, with subsequent single-stage surgery consisting of initial diagnostic laparoscopy to identify metastatic disease/resectability with selective cytologic washings prior to eventually undergoing curative intent esophagectomy. This is additionally evident when comparing stage migration outcomes between those with Siewert II and III, where Siewert III behaves more like primary gastric cancer, with a comparatively higher upstaging rate than seen in Siewert II tumors. This is also clearly evident in the literature, which has shown that the upstaging rate utilizing diagnostic laparoscopy with routine cytology is 10.9% for early stage gastric cancer (REF). Eliminating a preoperative staging diagnostic laparoscopy would have the benefit of reducing an extra operating room procedure prior to initiation of neoadjuvant chemotherapy or chemoradiation, thereby reducing hospital costs, potential procedural complications, and improving the time to initiate both neoadjuvant treatment in addition to eventual curative intent esophagectomy.

There are several limitations to this study, which was retrospective in nature and limited to a single tertiary referral center. Among the patients with Siewert II and III who underwent diagnostic laparoscopy, only 14 were upstaged, limiting the power for multivariable analyses when modeling the outcome of upstaging. However, this is the largest of all the regionalized referral centers for esophagectomy in the entire region, with more than 40 esophagectomies performed per year. Additionally, radiotherapy, which is part of our center’s treatment algorithm, was not able to be completely and accurately identified due to outsourcing of radiation oncology referrals depending on geographic region where the patient resided. We were able to accurately capture neoadjuvant chemotherapy data, which may play a larger role overall on distant recurrence and micrometastatic disease and hence more meaningful for our 5-year survival results. It is also important to note that while our study found statistically significant differences in delays to chemotherapy (42 vs. 37 days) and surgery (135 vs. 125 days) in those who received a diagnostic laparoscopy, the clinical significance of these findings may be put into question. We believe their clinical relevance may be more beneficial to patients for social and psychological reasons to expedite their treatment from their day of diagnosis. Furthermore, there are current data to suggest that a delay of more than 35 days after chemoradiation for an esophagectomy leads to increased anastomotic leaks.^22^ Additionally, although the cost-savings may be clinically evident from omitting a routine diagnostic laparoscopy for patients with Siewert II, our institution plans to perform a formal cost-effectiveness economic analysis in the future to corroborate these findings.

## Conclusions

Oncologic staging of Siewert II GEJ cancer remains challenging with no current census guidelines. Among all patients diagnosed with Siewert II GEJ cancer at our institution, patients undergoing diagnostic laparoscopy had increased time to initiate chemotherapy and definitive surgery in addition to having no improvement on mortality. Routine diagnostic laparoscopy with cytology yields a low upstaging rate in Siewert II GEJ CA while delaying time to definitive surgery. Expediting definitive surgery with selective biopsy in lieu of diagnostic laparoscopy may improve oncologic outcomes for Siewert II GEJ CA.
